# Specific Disruption of Tsc1 in Ovarian Granulosa Cells Promotes Ovulation and Causes Progressive Accumulation of Corpora Lutea

**DOI:** 10.1371/journal.pone.0054052

**Published:** 2013-01-15

**Authors:** Lin Huang, Zhen-Bo Wang, Zong-Zhe Jiang, Meng-Wen Hu, Fei Lin, Qing-Hua Zhang, Yi-Bo Luo, Yi Hou, Yong Zhao, Heng-Yu Fan, Heide Schatten, Qing-Yuan Sun

**Affiliations:** 1 State Key Laboratory of Reproductive Biology, Institute of Zoology, Chinese Academy of Sciences, Beijing, China; 2 Graduate School, Chinese Academy of Sciences, Beijing, China; 3 State Key Laboratory of Biomembrane and Membrane Biotechnology, Institute of Zoology, Chinese Academy of Sciences, Beijing, China; 4 Life Science Institute, Zhejiang University, Zhejiang Province, China; 5 Department of Veterinary Pathobiology, University of Missouri, Columbia, Missouri, United States of America; University of Connecticut, United States of America

## Abstract

Tuberous sclerosis complex 1 (*Tsc1*) is a tumor suppressor negatively regulating mammalian target of rapamycin complex 1 (mTORC1). It is reported that mice lacking *Tsc1* gene in oocytes show depletion of primordial follicles, resulting in premature ovarian failure and subsequent infertility. A recent study indicated that deletion of *Tsc1* in somatic cells of the reproductive tract caused infertility of female mice. However, it is not known whether specific disruption of *Tsc1* in granulosa cells influences the reproductive activity of female mice. To clarify this problem, we mated *Tsc1^flox/flox^* mice with transgenic mice strain expressing cyp19-cre which exclusively expresses in granulosa cells of the ovary. Our results demonstrated that *Tsc1^flox/flox^*; *cyp19-cre* mutant mice were fertile, ovulating more oocytes and giving birth to more pups than control *Tsc1^flox/flox^* mice. Progressive accumulation of corpora lutea occurred in the *Tsc1^flox/flox^*; *cyp19-cre* mutant mice with advanced age. These phenotypes could be explained by the elevated activity of mTORC1, as indicated by increased phosphorylation of rpS6, a substrate of S6 in the *Tsc1^flox/flox^*; *cyp19-cre* mutant granulosa cells. In addition, rapamycin, a specific mTORC1 inhibitor, effectively rescued the phenotype caused by increased mTORC1 activity in the *Tsc1^cko^* ovaries. Our data suggest that conditional knockout of Tsc1 in granulosa cells promotes reproductive activity in mice.

## Introduction

In mammals, folliculogenesis is strictly controlled by FSH and LH. FSH supports follicles to develop to the preovulatory stage, and the LH surge causes ovulation and rapidly initiates terminal differentiation of ovulated follicles into the corpora lutea [1], [2]. Numerous signaling pathways participate in these processes, such as phosphoinositide-3 kinase (PI3K), ERK1/2 and cAMP/protein kinase A pathways. These pathways coordinate expression of a huge number of genes in granulosa cells stimulated by FSH and LH [3].

Phosphoinositide-3 kinase (PI3K) signaling is a well known pathway, playing a vital role in many biological processes related to cancer, immunity, metabolism, and others [4]. FSH rapidly activates the PI3K pathway, initiating AKT phosphorylation. Activated AKT then phosphorylates its target proteins, FOXO1 (Forkhead winged helix box O1) and FOXO3 (Forkhead winged helix box O3) to control granulosa cell function and differentiation [3], [5], [6], [7]. *In vitro* experiments demonstrate that PI3K pathway mediated by FSH in granulosa cells is essential for differentiation and expansion of granulosa cells [6], [7], [8], [9]. In addition, oocyte-specific deletion of PI3K pathway members causes premature ovarian failure because of global primordial follicle activation [10], [11], [12], [13]. Moreover, conditional knockout of *Pten* in ovary granulosa cells promotes ovulation and causes progressive accumulation of corpora lutea [14]. These results indicate that the PI3K pathway is very important for ovarian functions.

In oocytes, the activation of S6-RPS6 by *Pten* deletion largely depends on mammalian target of rapamycin complex I (mTORC1) [12]. This indicates that mTORC1 is one critical downstream effector of the PI3K pathway [15]. As a serine/threonine kinase that regulates cell growth and proliferation by modulating processes such as ribosome biogenesis, protein synthesis and cell autophagy, the activity of mTORC1 is negatively regulated by a heterodimeric complex consisting of two proteins: TSC1 (hamartin) and TSC2 (tuberin) [15], [16]. *Tsc1* and *Tsc2* are two tumor suppressor genes, inactivating mutations in either of which explains genetically why patients suffer multiple tumors in various tissues and organs [17]. In cells, TSC1 and TSC2 form a heterodimeric complex, and TSC1 stabilizes TSC2 by protecting it from ubiquitination and degradation. The TSC1/2 complex controls cell growth, metabolism and proliferation by suppressing mTORC1 activation through a GTPase mechanisms [18], [19].

In order to determine the role of *Tsc1/2* in development in vivo, mouse models for studying function of *Tsc1* and *Tsc2* have been developed. Because of embryonic lethality caused by conventional deletion of *Tsc1* or *Tsc2,* conditional knockout of *Tsc1* and *Tsc2* in specific organs have been introduced via the *Cre-loxP* system [20], [21]. Specific deletion of *Tsc1* or *Tsc2* caused abnormalities in brain, heart, and kidney [22], [23], [24], [25]. In the ovary, oocyte specific disruption of either *Tsc1* or *Tsc2* leads to global activation of primordial follicles at the time of puberty, resulting in early follicle depletion and premature ovarian failure (POF) [26], [27]. So far, there are also data about deletion of *Tsc1* in somatic cells of the mouse reproductive tract. Disruption of *Tsc1* introduced by *Amhr2-cre* caused defects in ovarian folliculogenesis, compromised oocyte/embryo integrity, obstruction of oviduct and failure of implantation, resulting in female infertility [28]. As described above, it is not clear whether specific depletion of *Tsc1* in granulosa cells contributes to the fertility/infertility in the *Tsc1^flox/flox^*; *Amhr2-cre* female mice, because of the wide expression of *Amhr2-cre* in some somatic cells of the reproductive tract [28], [29].

In the current study, in order to investigate the role of *Tsc1* in granulosa cells in female reproductive activity, we used *cyp19-cre* to specifically delete *Tsc1* expression in granulosa cells [30]. Our results show that increased activity of mTORC1 in granulosa cells caused by *Tsc1* deletion does not cause sterility. On the contrary, *Tsc1* depletion improves reproductive capacity to some extent, stimulates folliculogenesis, and leads to progressive accumulation of corpora lutea.

## Materials and Methods

### Mice

*Tsc1^flox/flox^* mice were maintained with a mixed genomic background of 129S4/SvJae and C57/BL6 [20], and *cyp19-cre* mice were maintained with C57/BL6 genomic background [30]. *Tsc1^flox/flox^* mice were crossed with *cyp19-cre* mice to generate *Tsc1^flox/flox^*; *cyp19-cre* (*Tsc1^cko^*) mutant mice which are homozygous for the *Tsc1* floxed allele and heterozygous for *cyp19-cre*. Animals that are homozygous for *Tsc1* floxed allele and *cyp19-cre* negative were used as control mice. Mice were housed in 12-hour alternating light/dark cycles, with free access to water and food. All experiments were conducted with the approval of the Animal Research Committee of the Institute of Zoology, Chinese Academy of Sciences, China.

### Fertility Superovulation and Natural Ovulation Analysis

To evaluate the reproductive activity, six individually housed *Tsc1^flox/flox^* and *Tsc1^cko^* female mice at the age of 6 weeks were crossed to *Tsc1^flox/flox^* male mice with known fertility. The numbers of pups and litters were recorded up to 6 months. At the age of 23d, female mice of both genotype were injected with 5 IU of PMSG (Sansheng, Ningbo China) followed 48 h later with 5IU of hCG (Sansheng, Ningbo China) for superovulation analysis. For natural ovulation, female mice in estrus were mated with male mice. The next morning, female mice with plugs were euthanized, and fertilized eggs were separated from the oviduct and counted.

### Western Blot Analysis

Granulosa cells were collected from COC of six *Tsc1^flox/flox^* or *Tsc1^cko^* mice at the age of 23d after superovulation. Proteins extracted from cell lysis were quantified for western blot analysis. The primary antibodies used were: Tsc1, Tsc2, Akt, phospho-Akt (ser473), and phospho-rpS6 (ser240/4) from Cell Signaling Technology (USA), rpS6 from Bioworld (USA). β-Tubulin from Abmart (USA) was used as a loading control. Western blot were carried out according to the instructions by suppliers of the respective antibodies and viewed using molecular imager™ (Bio-Rad).

### Histological Analysis of Ovaries

Ovaries were fixed in 4% paraformaldehyde, dehydrated in a graded ethanol series, cleared in xylene, and embedded in paraffin wax. The paraffin-embedded ovaries were sectioned serially at 8 µm and stained with hematoxylin and eosin for histological analysis.

### Rapamycin Treatment

Rapamycin (LC Laboratories, Worburn, MA) was dissolved to 50 mg/ml in ethanol and diluted in a vehicle containing 0.25% Tween-20 and 0.25% polyethylene glycol in PBS. Mice were given intraperitoneal injection with either rapamycin (a daily dosage of 5 mg/kg body weight) or vehicle alone. *Tsc1^cko^* mice were injected daily from PD21 to PD42 and euthanized at PD43, then one ovary of each mouse was weighed, fixed, dehydrated and embedded for morphological analysis; the other one was lysed for western blot analysis. For superovulation analysis, *Tsc1^cko^* mice were intraperitoneally injected with rapamycin (a daily dosage of 3 mg/kg body weight) or vehicle from PD21 to PD42, followed by PMSG (Sansheng, Ningbo China) and hCG (Sansheng, Ningbo China) treatment.

### Statistical Analysis

All experiments were repeated at least three times for statistical analysis. For comparisons, means and standard deviations were calculated, and the difference between two groups was compared using student’s t-test. Difference was considered significant if *P*<0.05.

## Results

### Generation of Mice with Disruption of *Tsc1* in Granulosa Cells

**Figure 1:**
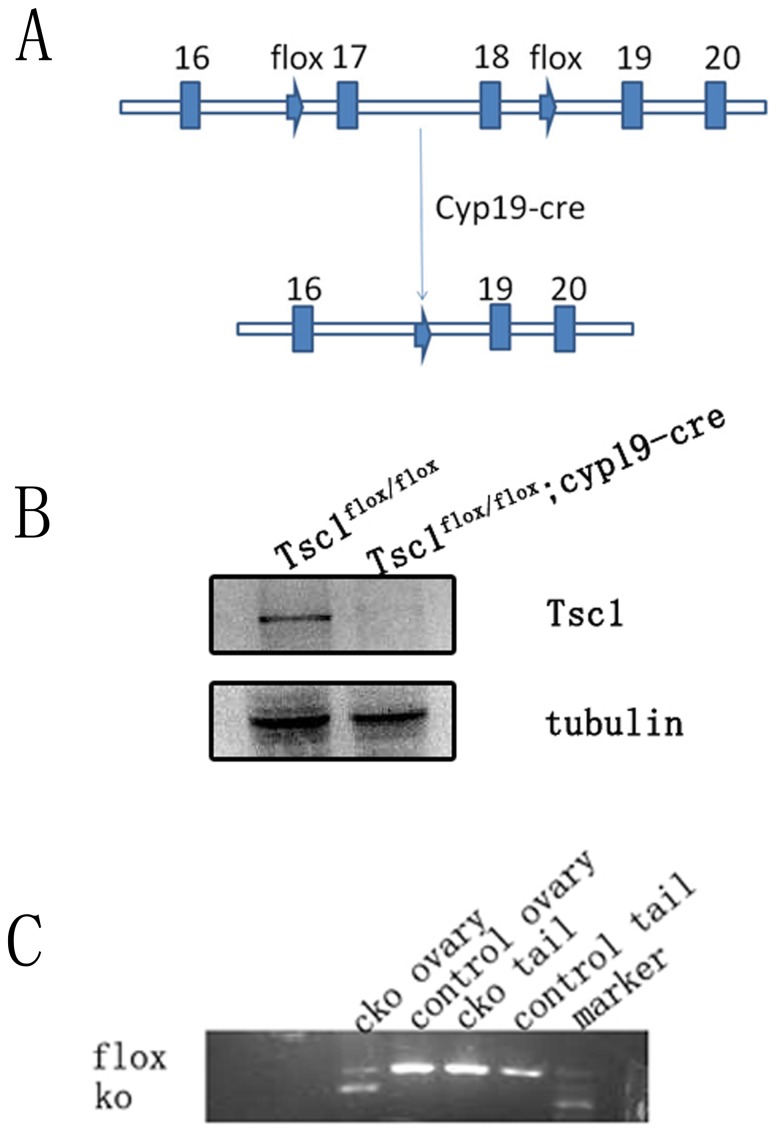
Generation of *Tsc1^cko^* mutant mice and characterization of *Tsc1* disruption by western blot and PCR analysis. (A) Schematic representation of deletion of *Tsc1* exon17 and exon18 by cyp19-cre mediated recombination in granulosa cells. (B) Granulosa cells were collected from COC of both *Tsc1^flox/flox^* mice and *Tsc1^cko^* mutant mice and lysed for western blot: *Tsc1* was almost absent in *Tsc1^cko^* granulosa cells, β-Tubulin was used as an internal control. (C) PCR analysis indicated that cyp19-cre mediated recombination of *Tsc1* exclusively occurred in *Tsc1^cko^* ovary.

To deplete *Tsc1* in granulosa cells, we crossed the *Tsc1^flox/flox^* mice [20] with transgenic mice carrying *cyp19* promoter-mediated Cre recombinase(*cyp19-cre*) [30] (Fig. 1A). The activity of *cyp19-cre* was detected in granulosa cells of all antral follicles and most luteal cells, but was low or nearly undetectable in granulosa cells of primordial and primary follicles [14], [30]. First we compared the expression of TSC1 protein in the granulosa cells of control and mutant ovaries, to confirm that the expression of TSC1 was diminished in the mutant granulosa cells. Cumulus granulosa cells were isolated from cumulus oocyte complexes (COC) from superovulated *Tsc1^flox/flox^* and *Tsc1^cko^* mice for western blot analysis (n = 6 per genotype). The results demonstrated that the Tsc1 protein was absent in *Tsc1^cko^* granulosa cells (Fig. 1B).

To confirm the recombination of the floxed alleles induced by *cyp19-cre*, we collected DNA from tails and ovaries of *Tsc1^flox/flox^* (control or WT) and *Tsc1^flox/flox^*: *cyp19-cre* (*Tsc1^cko^*) mice for PCR analysis. As expected, the DNA band representing depletion of the exon17 and exon18 of *Tsc1* only appeared in the amplicon of *Tsc1^cko^* mutant ovaries, demonstrating that recombination occurred exclusively in ovaries of *Tsc1^cko^* mice (Fig. 1C). All these results indicate that deletion of *Tsc1* is successful in granulosa cells of *Tsc1^cko^* mutant ovary where *cyp19-cre* is expressed.

### Increased Ovulation and Reproductive Activity in *Tsc1* Conditional Knockout Mice

**Figure 2:**
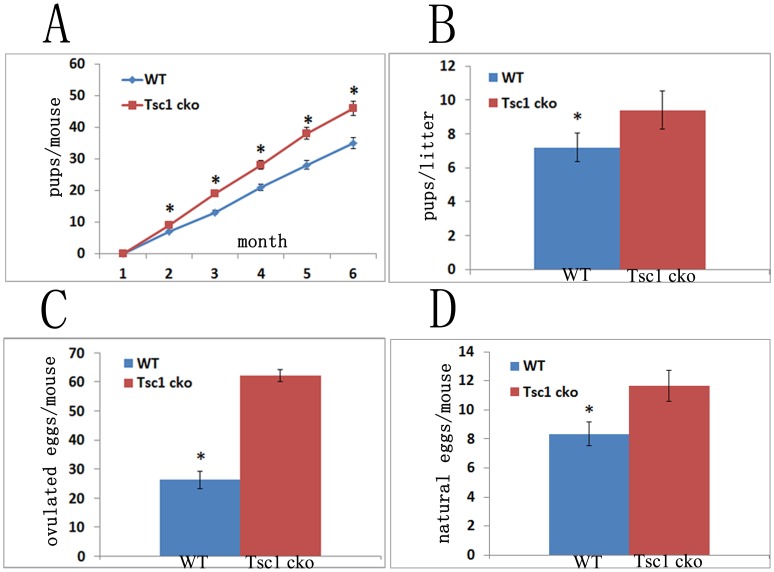
Evaluation of reproductive activity of *Tsc1^flox/flox^*;cyp19-cre mice. (A) *Tsc1^flox/flox^*; cyp19-cre mice produced more pups than wild type mice. (B) The average litter size of *Tsc1^flox/flox^*; cyp19-cre mice was bigger than that of the wild type. (C) *Tsc1^flox/flox^*; cyp19-cre mice ovulated more oocytes than wild type mice in superovulation analysis. (D) A little more oocytes were collected from the oviduct of *Tsc1^flox/flox^*; cyp19-cre mice than that from wild mice after natural ovulation and copulation.WT, wild type. *, *P*<0.05.

To test whether conditional knockout of *Tsc1* in granulosa cells affects the reproductive capacity of mutant mice, we mated *Tsc1^flox/flox^* and *Tsc1^cko^* female mice with *Tsc1^flox/flox^* male mice. Our observation demonstrated that *Tsc1^cko^* female mice were fertile, moreover, they even produced moderately more pups than *Tsc1^flox/flox^* control mice during a 6-month breeding period (n = 6 per genotype) (Fig. 2A). The average litter size of mutant mice was also larger than that of control mice. About 9 (9.4±0.50) pups per litter were born by *Tsc1^cko^* female mice, whereas the number of control mice was about 7 (7.2±0.37) (Fig. 2B). To confirm whether the increased number of pups born is attributed to elevated ovulation after *Tsc1* deletion, immature mice were primed with PMSG followed by hCG treatment after 48 hour for superovulation test. Thirteen hours after hCG injection, mice were euthanized, and their oviducts were removed for oocyte collection and analysis. Subsequent results indicated that *Tsc1^cko^* mutant mice ovulated more oocytes than control mice, accounting for the increased reproductive activity of *Tsc1^cko^* mutant mice (n = 4 per genotype) (Fig. 2C). More convincingly, more oocytes from the oviduct of *Tsc1^cko^* mutant mice than that from control oviduct were collected after natural ovulation and copulation (n = 3 per genotype) (Fig. 2D).

### *Tsc1* Conditional Knockout in Granulosa Cells Increased Folliculogenesis through Regulating the mTOR Pathway

**Figure 3:**
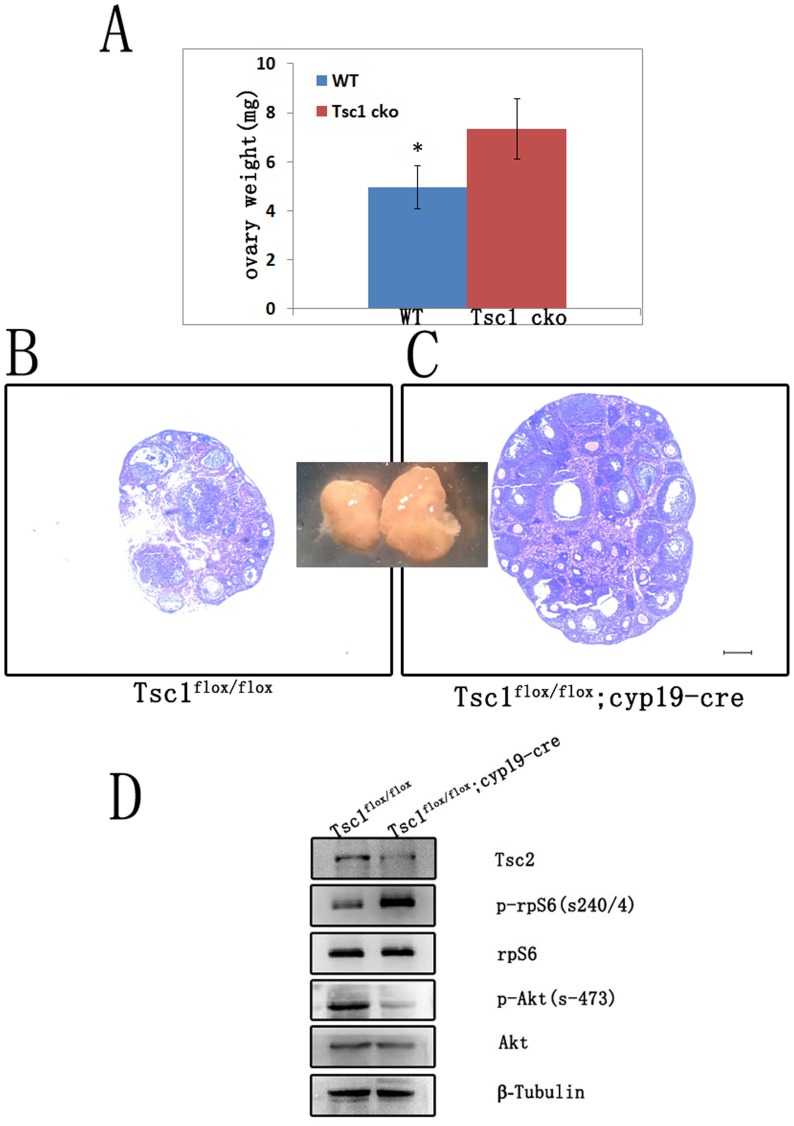
Conditional depletion of *Tsc1* in granulosa cells resulted in elevated mTORC1 activity, decreased mTORC2 activity and increased folliculogenesis. (A) Ovaries of *Tsc1^cko^* mutant mice were heavier than those of wild type mice. (B and C) Hematoxylin and eosin staining showed increased folliculogenesis in *Tsc1^cko^* mutant ovaries. (D) Western blot of granulosa cells from COC. After deletion of *Tsc1*, *Tsc2* was nearly absent; and the activity of mTORC1 was elevated, as indicated by increased phosphorylation of rpS6, a substrate of S6; meanwhile, activity of mTORC2 was down-regulated, as indicated by decreased phosphorylation of Akt. rpS6, Akt, and tubulin were used as control. Bar, 250 µm. *,*P*<0.05.

The observed change of *Tsc1* mutant mice may be ascribed to the increased follicle growth in the mutant ovary. To characterize the attribution, we collected ovaries from *Tsc1^flox/flox^* and *Tsc1^cko^* female mice at 6 weeks (n = 5 per genotype). As expected, *Tsc1^cko^* female mice had significantly heavier ovaries than *Tsc1^flox/flox^* control mice (Fig. 3A). Moreover, sections of the ovaries stained with hematoxylin and eosin demonstrated that there were more growing follicles and antral follicles in the ovaries of *Tsc1^cko^* mutant mice than that of *Tsc1^flox/flox^* mice (Fig. 3B and Fig. 3C).

In order to elaborate the molecular mechanism underlying the observed phenotypes in mutant ovary, we detected the expression level of several members regulated by *Tsc1*. Firstly, the expression of *Tsc2* was largely diminished after *Tsc1* deletion (Fig3D), consistent with the report that the function of *Tsc1* was to stabilize *Tsc2* [19]. mTOR falls into two distinct functional complexes, mTORC1 and mTORC2. While mTORC1 is rapamycin-sensitive, mTORC2 is rapamycin-insensitive [31], [32]. mTORC1 controls mRNA translation and promotes cell proliferation through phosphorylation of rpS6, a substrate of S6 kinase, which is a downstream target of mTOR kinase activity [15]. In contrast, mTORC2 is involved in cytoskeletal organization and phosphorylation of Akt [16]. We found that the level of phosphorylated rpS6 was elevated in *Tsc1^cko^* mutant granulosa cells compared to control cells, while the expression of rpS6 did not change significantly. On the contrary, phosphorylation of Akt decreased dramatically in *Tsc1* deleted granulosa cells while the level of Akt stayed constant (Fig. 3A). These results indicate that the activity of mTORC1 is elevated in mutant granulosa cells while mTORC2 activity is down-regulated.

### Progressive Accumulation of Corpora Lutea in *Tsc1* Conditional Knockout Mice

The corpus luteum is very important for the regulation of the estrous cycle and maintenance of pregnancy. After ovulation, the residual follicle undergoes luteinization to become the corpus luteum. If the oocyte is fertilized, the corpus luteum produces progesterone to maintain pregnancy. If fertilization does not occur the corpus luteum regresses, followed by a new estrous cycle [1], [2]. So the number of corpora lutea stays comparatively constant in every estrous cycle in the normal ovary.

**Figure 4:**
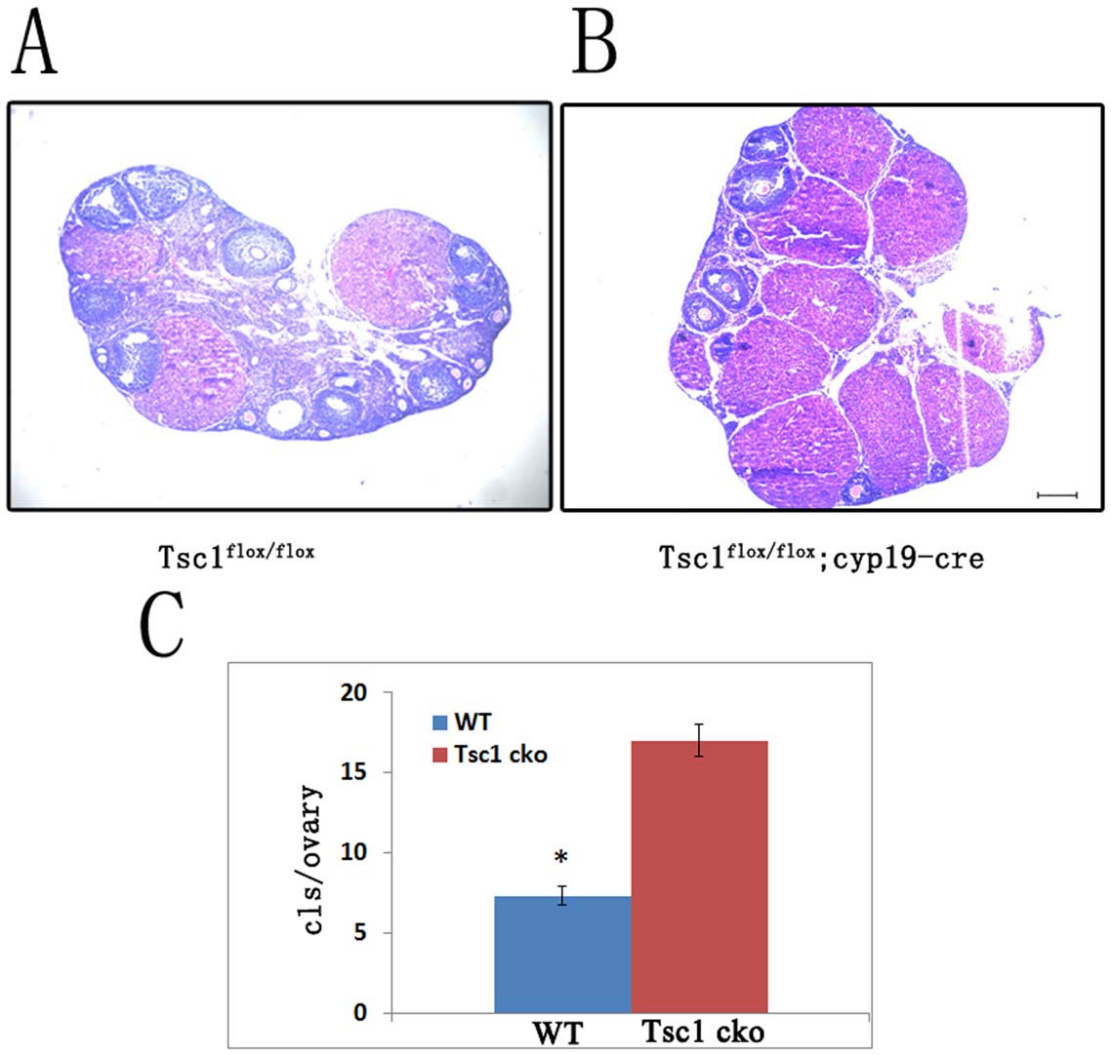
Progressive accumulation of corpora lutea. (A and B) Ovaries of *Tsc1^cko^* mutant mice and control mice at the age of 6 months were sectioned serially, and hemaxytoxylin and eosin staining showed accumulation of corpora lutea in *Tsc1^cko^* mutant ovaries compared with control ovaries. (C) Quantification of serial sections indicated that the numbers of corpora lutea in *Tsc1^cko^* mutant ovaries were about 3 times of that in control ovaries. Bar, 250 µm. *,*P*<0.05.

As reported for ovaries in which *Pten* was deleted in granulosa cells [14], we observed that progressive accumulation of corpora lutea occurred in the *Tsc1^cko^* mutant ovaries compared with control ovaries. There was no significant difference in the abundance of the corpora lutea in the mutant ovaries compared with control ovaries at the age of 6 weeks. It became gradually apparent that the ovaries of *Tsc1^cko^* mice contained more corpora lutea than those of control mice at 3 months of age. We ascertained the abundance of corpora lutea through histological analysis of 6-month-old Tsc1^cko^ mutant ovaries (Fig. 4A and Fig. 4B). We counted the overall corpora lutea of both control and mutant ovaries in serial sections (n = 3 per genotype). The results indicated that the number of corpora lutea in Tsc1^cko^ ovaries was about 3 times of that in normal cycling ovaries (Fig. 4C).

### Adjustment of Follicle Growth and Ovulation by Rapamycin

**Figure 5:**
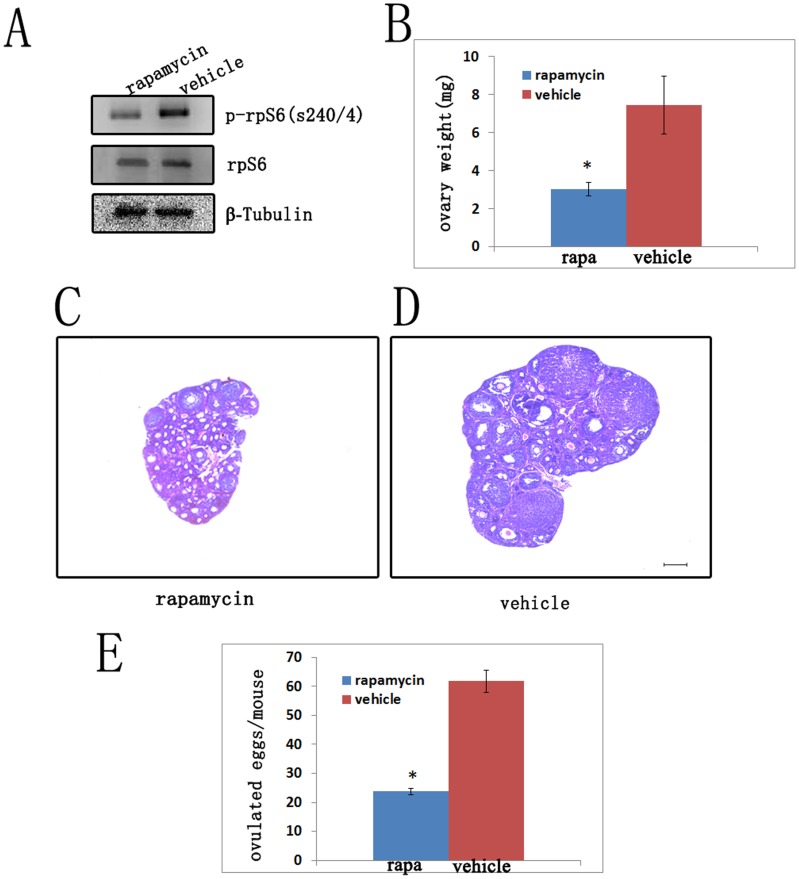
Adjustment of *Tsc1^cko^* mutant ovaries by postnatal rapamycin treatment. (A) Decreased activity of mTORC1 in *Tsc1^cko^* mutant ovaries after rapamycin treatment as indicated by down-regulated phosphorylated rpS6; rpS6 and tubulin were used as control. (B) The *Tsc1^cko^* mutant ovaries treated with rapamycin were lighter than those treated with vehicle. (C and D) Hemaxytoxylin and eosin staining showed that the folliculogenesis of *Tsc1^cko^* mutant ovaries declined after rapamycin treatment compared with controls. (E) The rapamycin treated *Tsc1^cko^* mutant mice ovulated less oocytes than *Tsc1^cko^* mutant mice treated with vehicle after superovulation. Bar, 250 µm. *,*P*<0.05.

It has been reported that the activity of mTORC1 is specifically inhibited by rapamycin [31]. So we treated the *Tsc1^cko^* mutant mice with rapamycin from post-natal day (PD) 21 to PD42, to ascertain whether it was the elevated activity of mTORC1 that promoted the follicular growth and ovulation. After rapamycin treatment, the expression level of phosphorylated rpS6 dramatically decreased compared with that of the group which was treated with vehicle (Fig. 5A).

Our observation also showed that the ovaries are smaller and lighter, containing less antral follicles in *Tsc1^cko^* mutant mice treated with rampamycin (Fig. 5B and Fig. 5C). In comparison, the ovaries of *Tsc1^cko^* mutant mice treated with vehicle were much larger and contained more antral follicles (Fig. 5B and Fig. 5D) (n = 3 per case). Moreover, the vehicle-treated mutant mice ovulated more oocytes than rapamycin-treated mutant mice in superovulation analysis (Fig. 5E) (n = 4 per case). These results indicate that rapamycin effectively rescued the phenotype caused by increased mTORC1 activity in the *Tsc1^cko^* ovaries. Our results clearly confirm that it is the elevated activity of mTORC1 that accounts for the increased follicular growth in the ovary of *Tsc1^cko^* mutant mice.

## Discussion

The tuberous sclerosis complex (TSC) is a multisystem, autosomal dominant disorder affecting both children and adults with a rate of one in 6000. TSC is characterized by developing benign tumors in various organs such as kidney, heart, brain, and others. Genetic analysis indicates that TSC patients carry mutations in either the harmatin (*Tsc1*) or tuberin (*Tsc2*) genes [17]. *Tsc1* and *Tsc2* work as heterodimers in cells. They control cell proliferation and survival by regulating the activity of mTOR and playing a vital role in many signaling cascades [18]. Indeed, conditional disruption of *Tsc1* or *Tsc2* in brain, kidney or heart causes corresponding abnormalities because of dysregulation of mTOR activity, and all the induced pathologies can be rescued by rapamycin, a specific inhibitor of mTOR [22], [23], [24], [25].

In ovaries, specific deletion of *Tsc1* or *Tsc2* in oocytes leads to primordial follicle depletion, causing premature ovarian failure [26], [27]. Conditional knockout of *Tsc1* in somatic cells of the reproductive tract results in infertility in female mice. It is noteworthy that Amhr2-cre previously used is not only expressed in granulosa cells, but also in the oviduct and uterus, so it is not confirmed whether specific deletion of *Tsc1* in granulosa cells results in female infertility or not [4]. In the current study, we deleted *Tsc1* exclusively in granulosa cells to clarify the function of *Tsc1* in ganulosa cells by using *cyp19-cre* transgenic mice because of its specific expression in the granulosa cells of the ovary [14], [30]. Our results demonstrate that disruption of *Tsc1* in granulosa cells does not contribute to female sterility. On the contrary, *Tsc1^cko^* mutant mice in our study breed more pups than control mice to some extent. This may be attributed to the increase in oocytes ovulated in cyp19-cre mediated mutant mice. Moreover, because *Tsc1^cko^* mutant mice give birth to pups even at the age of 6 months, so we do not believe that premature ovarian failure occurs in the female knockout mice.

Unlike mutation of *Tsc1* in other organs, we observed no tumors in *Tsc1^cko^* mutant ovaries. Interestingly, we found that corpus luteum progressively accumulated in the *Tsc1^cko^* mutant ovaries compared with control ovaries, which was identical to the ovaries in which *Pten* was conditionally deleted in granulosa cells [14]. However, corpora lutea that had prolonged lifespan caused by dysregulated activity of mTORC1 after loss of *Tsc1* appeared to have no impact on the steroidogenic activity, because *Tsc1^cko^* mutant mice had normal reproductive cycles as control mice (Fig. 2A), indicating that the function of the corpora lutea in *Tsc1^cko^* mutant ovaries was not altered. More detailed research is needed to investigate why the corpora lutea with extended lifespan does not have prolonged steroidogenic activity.

We observed in our study elevated activity of mTORC1 (represented by increased phosphorylation of rpS6) and decreased activity of mTORC2 (represented by decreased phosphorylation of Akt), both of which may account for the increased folliculogenesis and ovulation. A previous study reported that rapamycin is a specific inhibitor of mTORC1 [31]. In order to determine whether the observed increased folliculogenesis and ovulation were mTORC1- dependent after loss of *Tsc1*, we tried to rescue the phenotypes using rapamycin. As expected, rapamycin could rescue the folliculogenesis and ovulation. This result confirms that deletion of *Tsc1* leads to the phenotypes in *Tsc1^cko^* mutant mice through stimulation of mTORC1.

In summary, the present study shows that depletion of *Tsc1* in granulosa cells does not cause infertility in mice, but improves the reproductivity by stimulating folliculogenesis, ovulation and progressive accumulation of corpora lutea via increased activity of mTORC1. Although the phenotypes observed in our study were relatively mild, which may be attributed to preferential expression of cyp19-cre in the granulosa cells of antral follicle [30], our data still offer physiological and clinical implications of *Tsc1* for human ovarian development and pathology.
